# Trace-Forward Investigation of Mice in Response to Lymphocytic Choriomeningitis Virus Outbreak

**DOI:** 10.3201/eid2010.130861

**Published:** 2014-02

**Authors:** Laura Edison, Barbara Knust, Bret Petersen, Julie Gabel, Craig Manning, Cherie Drenzek, Ute Ströher, Pierre E. Rollin, Douglas Thoroughman, Stuart T. Nichol, the Multistate LCMV Outbreak Working Group

**Affiliations:** Centers for Disease Control and Prevention, Atlanta, Georgia, USA (L. Edison, B. Knust, B. Petersen, C. Manning, U. Ströeher, P.E. Rollin, D. Thoroughman, S.T. Nichol);; Georgia Department of Public Health, Atlanta (L. Edison, J. Gabel, C. Drenzek);; Kentucky Department for Public Health, Lexington, Kentucky, USA (D. Thoroughman)

**Keywords:** animals, mice, humans, lymphocytic choriomeningitis virus, arenavirus, zoonoses, viruses, multistate outbreaks

## Abstract

During follow-up of a 2012 US outbreak of lymphocytic choriomeningitis virus (LCMV), we conducted a trace-forward investigation. LCMV-infected feeder mice originating from a US rodent breeding facility had been distributed to >500 locations in 21 states. All mice from the facility were euthanized, and no additional persons tested positive for LCMV infection.

Lymphocytic choriomeningitis virus (LCMV), a rodent-borne arenavirus, causes inapparent infection in mice but can cause febrile illness, aseptic meningitis, encephalitis, and severe birth defects in humans (www.cdc.gov/ncidod/dvrd/spb/mnpages/dispages/lcmv.htm) (*1*). LCMV also can cause disseminated disease with substantial mortality among infected organ transplant recipients (*2*). The reservoir is the common house mouse, *Mus musculus*, but other rodents can become infected and transmit infection to humans. LCMV is endemic among house mice throughout the world, with antibody seroprevalence of 5%–13% in the United States (*3*). LCMV is easily maintained after being introduced into a captive mouse population because mice can persistently shed the virus. LCMV can be transmitted to humans through direct or aerosol contact with urine, feces, or saliva of infected rodents; through transplantation of infected organs; and from mother to fetus (*1*). Sporadic cases occur from exposure to peridomestic house mice, and outbreaks from exposure to infected rodents, particularly hamsters, kept as pets or used for laboratory experimentation have been reported (*2*,*4*–*7*). No outbreaks have been linked to contact with frozen mice.

During summer 2012, state and local agencies and the Centers for Disease Control and Prevention (CDC; Atlanta, GA, USA) investigated an outbreak of LCMV in the United States. A total of 31 (32%) of 97 tested employees of 3 rodent breeding facilities were infected because of the likely introduction of LCMV into the captive breeding population by wild mice. LCMV aseptic meningitis was diagnosed in 4 employees, and diagnostic testing of the breeding population identified LCMV infection among mice but not rats; no hamsters were bred at the facility (facility A in [*8*,*9*]). All mice originating from this captive breeding population were considered potentially infected and had been distributed to rodent purchasing facilities in multiple states by an Indiana rodent distributor, facility B. We describe the trace-forward investigation of live mice distributed by facility B and the public health measures taken to prevent additional human LCMV infections.

## The Study

**Figure 1:**
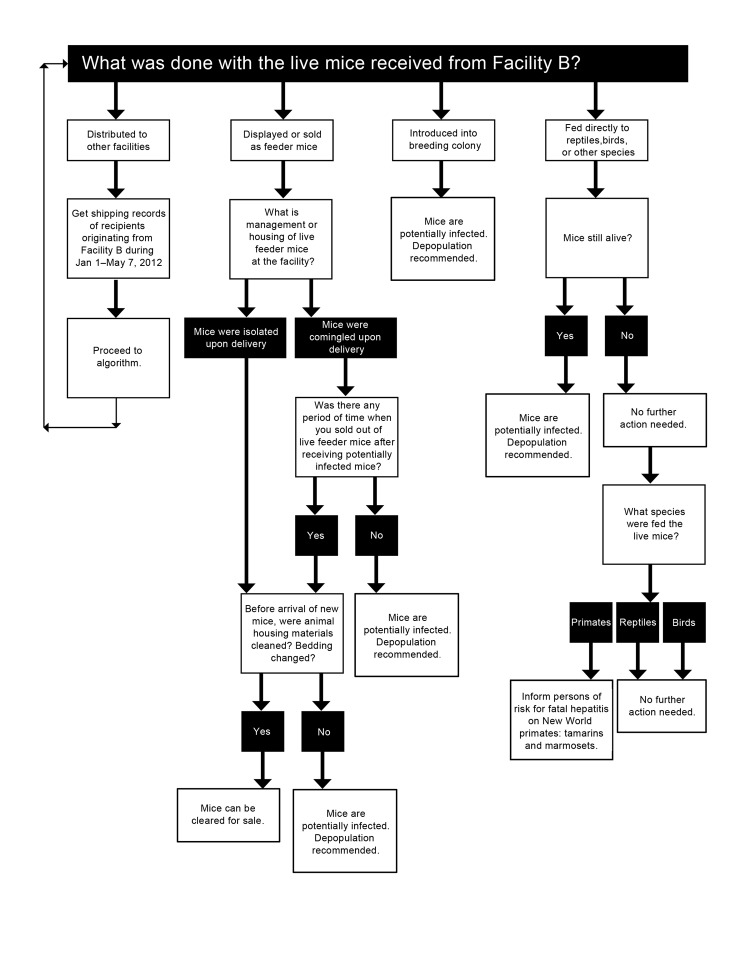
Algorithm used to determine whether mice were potentially infected with lymphocytic choriomeningitis virus (LCMV) during a multistate investigation, United States, 2012. This algorithm was used to determine whether 1) potentially infected mice remained at the facilities being assessed, 2) mice from the original shipment remained, 3) offspring from these mice remained, or 4) shipments of mice had been comingled or had shared equipment with mice from the original shipment. LCMV is easily maintained in a mouse colony, and a clear break among the population (i.e., a time when no remaining mice are maintained and equipment is disinfected) is necessary to ensure that no ongoing infection continues.

During July and August 2012, investigators from CDC’s Viral Special Pathogens Branch reviewed shipping records from facility B and subsequent distributors and notified health departments in states that had received potentially infected mice during January 1–May 7, 2012; frozen mice were considered a low public health risk and were not traced. Health departments were provided with a list of facilities that had purchased these mice, educational resources about LCMV, and an algorithm to determine whether potentially infected mice remained at these purchasing facilities resulting from the presence, comingling, or breeding of these mice, which would maintain LCMV among the mouse population (Figure 1). As a result of varying state statutes concerning regulation and licensing of pet stores and animal breeders or distributors, the government agencies that had jurisdiction to perform these investigations included local and state departments of public health, environmental health, food safety, and agriculture.

State investigators interviewed purchasing facility managers by telephone, mail, email, or in person to determine whether potentially infected mice remained on the premises and to encourage euthanization of these mice. Interviews also assessed whether pregnant, ill, or immunocompromised employees might have been exposed to LCMV by directly handling potentially infected mice or bedding or equipment used for the mice. Because of risk for severe disease, facility managers were asked to offer serologic testing to these employees for LCMV IgM and IgG, which was performed by CDC by using ELISA as described (*10*). No additional case-finding activities were conducted. Because of resource limitations, diagnostic testing of live mice at purchasing facilities was not conducted.

**Figure 2:**
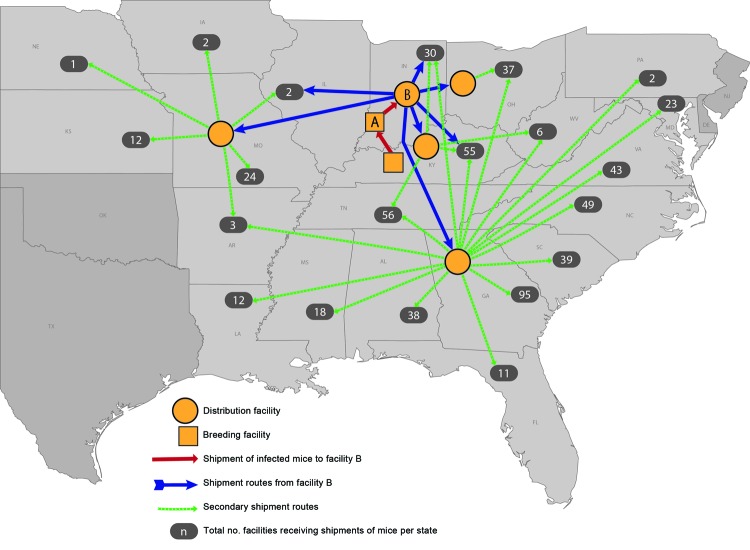
The distribution of mice potentially infected with lymphocytic choriomeningitis virus originating from facility A to ≈500 pet stores and other animal facilities in 21 states, United States, 2012.

Reviews of shipping records indicated that ≈304,000 live mice distributed by facility B were shipped to 561 purchasing facilities: 543 pet stores, 11 breeders or distributors, and 7 zoos or aquariums in 21 states, potentially exposing thousands of employees and pet store mouse purchasers to LCMV. Facility B had shipped mice to 4 subsequent distributors; the largest was located in Georgia, and it had shipped >183,000 mice to 420 purchasing facilities in 16 states (Figure 2). Interviews of facility managers at purchasing facilities revealed that 48% still had potentially infected mice; >10,000 mice were subsequently euthanized. The most common reason for still having potentially infected mice was comingling of rodent shipments, followed by breeding or still having mice from the original shipments.

Serologic testing was performed on blood samples from 34 pet store or zoo employees from 6 states who self-identified as pregnant or ill, were potentially exposed to LCMV, and agreed to serologic testing. Fourteen were pregnant; 1 had aseptic meningitis; and 23 reported nonspecific symptoms including fever, headache, body aches, cough, and vomiting. All persons tested were negative for antibodies against LCMV.

## Conclusions

These captive feeder mice had a wide and complex distribution chain, potentially exposing thousands of persons to LCMV. No additional human cases were identified after distribution of these mice; none of the pet store or zoo employees tested had serologic evidence of infection. Although no additional human cases were identified, euthanasia of all potentially infected rodents was recommended to mitigate potential risk.

Wild mice that access captive breeding populations are often the source of infection of captive rodent populations (*8**,**11*). After being introduced, LCMV transmission is easily maintained among mouse colonies and is difficult to recognize because mice do not appear ill. Because persistently infected mice pass infection to their offspring, the number of infected mice in a breeding colony can quickly multiply. Mice can be persistently infected without having serologic evidence of infection (*12*); thus, LCMV can be missed by serologic screening alone. Therefore, preventing introduction of the virus into breeding colonies, depopulation of infected rodents, and correct use of personal protective equipment are the most efficient ways to mitigate human exposure. We recommend preventive measures at each point in the distribution process, both domestically and abroad (Table) (*13*–*15*). More research is needed to develop methods for detecting LCMV in rodents at distributors and pet stores.

**Table:** Recommendations for the prevention and control of lymphocytic choriomeningitis virus among rodents and persons who handle them

Preventing lymphocytic choriomeningitis virus (LCMV) from being introduced into a rodent breeding colony
• All rodents introduced into the breeding colony facility should come from a facility with a biosecurity and monitoring program for LCMV in place.
• Contact between wild mice and breeding colony animals should be prevented through exclusion and by trapping of escaped and feral mice; rodent traps should be placed at the perimeter of the facility, in rodent rooms, and in areas where feed is stored.
• Any feral rodents or colony rodents that escape should be removed and euthanized.
• Testing for LCMV should be included in the routine health monitoring for the colony.
Preventing the spread of LCMV within a rodent breeding colony
• Movement of animals between rooms should be restricted; replacement breeding animals remain within the room they were born, and animals only leave rooms to be removed from the facility.
• Equipment used in handling used cages or bedding should not be shared between rooms or used to handle clean cages or bedding, and such equipment should be disinfected regularly.
• Employees should wear waterproof washable or disposable footwear that can be cleaned between rooms, and they should wear designated coveralls or laboratory coats for each building or room. Footwear should be disinfected before exit and entry into each room.
• After LCMV is discovered, all rodents in the affected rooms or colony should be euthanized.
Preventing infection or the spread of infection at the rodent distributor or pet store
• Rodents should be purchased only from suppliers with biosecurity and monitoring programs.
• Comingling of rodents from different shipments and between rodent species should be prevented.
• Contact between wild mice and captive rodents should be prevented through exclusion and by trapping of escaped rodents and wild mice; rodent traps should be placed at the perimeter of the facility, in rodent rooms, and in areas where feed is stored.
• Equipment used in handling used cages or bedding should not be shared between shipments of rodents or used to handle clean cages or bedding, and such equipment should be disinfected regularly.
Preventing infection among persons who handle rodents
• Employees who handle rodents should be educated about the risk for LCMV, and educational material should be distributed at the point of purchase in pet stores.
• Gloves should be used during handling of live or frozen rodents, used bedding, and dirty cages; hands should be promptly washed when gloves are removed.
• Pregnant and immunocompromised persons should be advised not to directly handle rodents or clean cages.
• Employees should not eat, drink, or smoke in rodent rooms.
Handling rodents with known LCMV infection
• A respirator with a filter of >N95 rating, filtering face piece, elastomeric half or full mask, or powered air-purifying respirator should be worn; respiratory capacity testing and fit testing are necessary for all persons wearing such gear.
• Gloves, waterproof and washable footwear, and coveralls should be worn; disinfectant should be used to clean the external surfaces of the protective gear, and workers should wash their hands after removing gloves.

Our investigation had several limitations. Employees tested were a fraction of those who had had contact with potentially infected mice. Also, pet store mouse purchasers and purchasing facility employees were difficult to contact, and no pet store customers were tested. Thus, the true number of infected persons is unknown.

Rodent breeders and distributors can fall through a regulatory gap in the United States. Frozen feeder mice are considered pet food and can be regulated by the Food and Drug Administration (FDA), but neither FDA nor the US Department of Agriculture has the authority to regulate live mice and rats because they are not regulated under the Animal Welfare Act (7 CFR 2132, May 13, 2002, www.aphis.usda.gov/animal_welfare/downloads/awa/awa.pdf) and the Food, Drug and Cosmetics Act (21 CFR 500, April 1, 2012, www.accessdata.fda.gov/scripts/cdrh/cfdocs/cfcfr/CFRSearch.cfm?CFRPart=500&showFR=1). Therefore, regulatory authority falls to the states, which have an array of regulations governing the handling, breeding, and distribution of rodents, including the licensing of pet breeders and distributors (*15*; Thomas Edling, pers. comm.), as was evident in this trace-forward investigation. Because of the lack of consistent regulation, we recommend that state and federal partners and rodent industry advisory groups work with breeders, distributors, and pet stores to increase awareness of LCMV infection and implement recommended best practices (Table) to prevent introduction of LCMV into captive rodent populations, prevent subsequent dissemination of potentially infected rodents, and reduce the potential for human exposure and disease among employees and consumers of pet stores and rodent breeding facilities.

Technical Appendix Multistate LCMV outbreak, United States, July 2012: recommendations for trace-back and safe disposal of potentially LCMV-infected mice.
