# Supplementary material for: Trace-Forward Investigation of Mice in Response to Lymphocytic Choriomeningitis Virus Outbreak
Source: Emerg Infect Dis. 2014 Feb;20(2):291–5. doi: 10.3201/eid2010.130861 (PMC3901476; doi:10.3201/eid2010.130861)
Supplement: Technical Appendix — Multistate LCMV outbreak, United States, July 2012: recommendations for trace-back and safe disposal of potentially LCMV-infected mice. [file 13-0861-Techapp-s1.pdf]

Article DOI: http://dx.doi.org/10.3201/eid2002.130861

Trace-Forward Investigation of Mice in Response to Lymphocytic Choriomeningitis Virus Outbreak

Technical Appendix

Multistate Lymphocytic Choriomeningitis Virus (LCMV) Outbreak July 2012: Recommendations on Trace-back and Safe Disposal of Potentially LCMV-infected Mice

Introduction

The Centers for Disease Control and Prevention (CDC) is conducting an ongoing investigation of an outbreak of lymphocytic choriomeningitis virus (LCMV) in employees at a large commercial rodent breeding facility in Indiana, USA. During CDC's investigation, it was learned that live, potentially LCMV-infected mice were shipped from the Indiana facility to purchasers, including pet stores, in several states up through May 7, 2012. These potentially LCMV-infected mice can infect other rodents and also pose a health risk to persons who come into contact with them. LCMV infection in people produces symptoms ranging from mild illness to aseptic meningitis. Pregnant women may pass infection to the fetus, resulting in birth defects. Immunocompromised persons are also at increased risk of developing severe disease.

Recommendations

CDC recommends that euthanasia and safe disposal of all potentially LCMV-infected rodents (mice, hamsters, gerbils, guinea pigs) be undertaken. The animals that are potentially infected with LCMV include all rodents originating from the implicated facility in Indiana and any rodents that have been commingled with these mice. State and local health authorities will determine the disposition of animals at locations in the individual states that have received shipments of potentially LCMV-infected mice.

Details describing the processes for safe euthanasia and disposal are described below.

This document provides CDC responses to several groups affected by this outbreak:

- Rodent distribution facilities that receive, house, and ship rodents (mice, hamsters, and/or gerbils);
- Pet stores that receive live rodents and then directly retail to the public;
- Zoos, which receive live rodents for feeding to reptiles, raptors, and other animals;
- State and county public health staff;
- Reptile owners; and
- Rodent breeders.

Q&A

Q: Given that these mice were shipped back in May, why is it important to euthanize mice at our facility now? Is there possible ongoing transmission occurring at our facility?

A: Euthanasia should occur if LCMV-infected mice have commingled with a facility's other rodent population(s) because LCMV can spread through direct contact between rodents or through contaminated bedding or housing. When a mouse or other rodent becomes infected, it may shed the virus for months in its urine and saliva, showing no signs of illness. Facilities that have effectively isolated newly arrived rodents from existing populations do not have to euthanize those rodents in the existing populations.

Q: How do we distinguish between rodents that need to be euthanized and safely disposed of and those that are not potentially infected?

A: There are 3 categories of rodents that should be identified for euthanasia and safe disposal:

- Mice shipped from the implicated facility in Indiana before the quarantine was written on May 7; this is the originally LCMV-infected population, consisting only of white mice;
- Rodents that have been commingled with the above population; these could include mice or other rodents at distribution facilities or pet stores, with which the potentially LCMV-infected mice were housed;

- Rodents that had shared equipment with the potentially LCMV-infected mice (e.g., cages, water bottles, holding containers) that were not cleaned with soap and water and/or disinfectant before being moved between animals.

Q: Given that LCMV might be present in rodents at our facility, what precautions should be taken by employers and employees?

A: Employers should educate all employees about risks of exposures and potential health effects related to work with rodents. During a known outbreak (such as the current situation), the education should be reinforced for groups at increased risk; women who are or may become pregnant should be educated about risks to the fetus, and all staff should be educated about increased health risks for persons who may be immunocompromised. If any staff member is pregnant or immunocompromised and directly handled the potentially infected rodents, they should be tested to determine if they have been exposed to LCMV. Any workers handling potentially LCMV-infected rodents should wear proper personal protective equipment (PPE) (see next question).

Q: What is the proper PPE to handle potentially LCMV-infected rodents?

A: Proper PPE for any persons handling potentially LCMV-infected rodents, cleaning their cages, or handling their bedding materials includes latex or nitrile gloves, an N95 filtering face piece respirator or higher level particulate respirator, and eye protection. Because broken skin can be a portal for entry of the virus, breaks in the skin should be covered. Efforts should be made to minimize the generation of aerosols while cleaning cages. All respirator users should be fit-tested before use, and respirators should be used within the context of a complete respirator program that meets the requirements in the Occupational Safety and Health Administration (OSHA) respirator standard (29 CFR 1910.134). Hands should be washed with soap and water or an alcohol-based hand sanitizer after removal of gloves. A lab coat, coverall, or work shirt that can be removed after exposure to the animals and laundered is also recommended.

Q: Who will perform the euthanasia and safe disposal of the rodents?

A: Each state or local health department will determine who will be designated to do this.

Q: What is the proper method for euthanasia and disposal of potentially LCMV-infected mice?

A: The best method for euthanasia is one that minimizes direct handling of the mice to reduce the risk of LCMV transmission to the person. In this case, CDC recommends using CO_2 gas or another anesthetic agent.

Once the rodents have been euthanized, they should be disposed of as follows: Wearing gloves, spray the rodents with a commercial disinfectant or with 5% bleach solution and let stand for 5 minutes. At the end of 5 minutes, pack the rodents into a plastic trash bag. Close the top of the bag. The bags of dead/disinfected rodents can be buried at a depth of $\geq$3 feet, burned, or double-bagged and transported to a landfill. Those engaged in rodent disposal activities should wear gloves when handling closed bags and wash their hands after removing the gloves.

Q: Can our mice be tested to determine if they are infected?

A: Yes, there is commercial testing available to detect LCMV; however, the animals must be euthanized to determine whether they are actively infected with the virus. If only blood tests are performed, there is the possibility that infection can be missed. In most cases, the cost of testing exceeds the value of the animals in question.

Q: Is it possible for an LCMV-infected mouse to infect other rodents at our facility, like hamsters and gerbils?

A: Yes—any rodents that were in direct contact with potentially infected mice or shared the same housing (cage, water bottle, feed dish) without disinfecting between uses could become infected, and these animals may potentially pass the infection to people.

Q: We received frozen mice in addition to live mice from the implicated facility. Are these safe to use?

A: Although frozen mice pose less risk of LCMV exposure than live mice, persons should wear gloves when thawing and handling them and wash their hands afterward. Frozen mice should be fed to animals whole.

Q: Can the reptiles or birds that consumed these infected mice develop asymptomatic LCMV infection, symptomatic LCMV disease, or be able to transmit LCMV to their human handlers through contact with them or their feces?

A: Because LCMV infection in animals eating feeder mice has not been widely studied, we do not know whether reptiles or birds consuming such animals can themselves develop an infection.

There is, however, a risk for challitrichid primates (marmosets and tamarins) who consume infected mice to develop infection, which results in hepatitis with high fatality. If there have been any unusual recent illnesses or deaths in the animals that were fed live mice, CDC would be interested in testing any available specimens.

Q: Is CDC willing to conduct testing for LCMV for people who were potentially exposed to the virus?

A: Yes. CDC is especially concerned about pregnant or immunocompromised persons who may have come in contact with the mice and would recommend testing these people because of the risk for more severe disease and congenital illness. CDC would also be interested in testing any persons who had exposure to the potentially LCMV-infected mice and were treated with symptoms of meningitis.

More information about LCMV is available at
www.cdc.gov/ncidod/dvrd/spb/mnpages/dispages/lcmv.htm
